# Social Media engagement and perceived social isolation among older adults in a changing digital environment: evidence from HINTS 2022–2024

**DOI:** 10.3389/fdgth.2026.1883306

**Published:** 2026-08-18

**Authors:** Jingjing Gao

**Affiliations:** Department of Management, Policy, and Community Health, University of Texas Health Science Center at Houston School of Public Health, Houston, TX, United States

**Keywords:** artificial intelligence, digital divide, digital health, healthy aging, hints, older adults, perceived social isolation, PROMIS social isolation scale

## Abstract

**Background:**

The rapid expansion of social media and emerging artificial intelligence (AI)-mediated digital environments has transformed how older adults communicate, access information, and engage with online communities. Social media platforms may provide opportunities for social connectedness and emotional support, particularly among older adults who face mobility limitations, retirement-related social changes, or reduced in-person interactions. At the same time, increased reliance on digital environments may also contribute to perceived social isolation through social comparison, exposure to misinformation, fragmented online interactions, or difficulties navigating evolving technologies. Despite growing interest in digital health and aging, limited research has examined the relationship between social media use and perceived social isolation among older adults during the emerging AI-enabled digital era.

**Objective:**

This study aimed to examine the association between social media engagement and PROMIS social isolation T-scores among U.S. adults aged 65 years and older, while accounting for sociodemographic characteristics, digital access-related factors, and survey cycle using nationally representative data from the Health Information National Trends Survey (HINTS) 2022 and 2024 cycles. The study also conducted exploratory interaction analyses by age and survey cycle to assess whether associations varied across older age and between HINTS cycles.

**Methods:**

This cross-sectional study utilized pooled nationally representative data from HINTS 7 cycles conducted in 2022 and 2024. The analytic sample included adults aged 65 years and older. Perceived social isolation was assessed using the PROMIS Social Isolation Scale T-score (PROMIS_Isolation_t), a validated composite measure of perceived social isolation derived from four survey items assessing feelings of isolation, exclusion, and social disconnection. Social media use was measured using self-reported engagement with social networking platforms. Survey-weighted descriptive statistics and multivariable regression analyses were conducted to evaluate the association between social media use and perceived social isolation while adjusting for age, sex, race/ethnicity, education, household income, and survey cycle.

**Results:**

Older adults who reported social media use demonstrated differing levels of perceived social isolation compared with nonusers. Social media engagement was positively associated with higher PROMIS social isolation T-scores after adjustment for age, sex, race/ethnicity, education, household income, and survey cycle. Higher household income was generally associated with lower PROMIS social isolation T-scores. Sensitivity analyses using binary social media engagement indicators showed consistent associations across engagement behaviors. Differences were particularly observed among individuals with lower educational attainment and limited digital literacy. Findings also suggested that the evolving AI-mediated digital environment may influence how older adults interact with online social platforms and perceive social connectedness.

**Discussion:**

The findings suggest that social media use among older adults may have both beneficial and adverse implications for perceived social isolation within the emerging AI-mediated digital environment. Social media platforms may facilitate communication, information exchange, and social participation, potentially reducing perceived social isolation for some older adults. However, unequal digital literacy, technological complexity, and potentially harmful online experiences may contribute to persistent or worsening perceptions of isolation among vulnerable populations. These findings highlight the importance of promoting digital inclusion and age-friendly online environments as part of broader healthy aging and public health strategies.

**Conclusion:**

Social media use is increasingly embedded within AI-mediated digital environments and may play an important role in shaping perceived social isolation among older adults in the United States. Public health interventions aimed at improving digital literacy, equitable technology access, and supportive online engagement may help reduce perceived social isolation and promote healthy aging among older populations.

## 1 Introduction

Perceived social isolation and social isolation are increasingly recognized as critical public health concerns among older adults in the United States. A growing body of literature has linked perceived social isolation to numerous adverse physical and mental health outcomes, including depression, anxiety, cognitive decline, cardiovascular disease, frailty, reduced quality of life, and premature mortality (1–7). Older adults may be particularly vulnerable to social isolation due to retirement, bereavement, chronic disease burden, mobility limitations, and reduced opportunities for in-person social interaction (8–12). As the U.S. population continues to age, understanding factors associated with perceived social isolation among older adults has become an important public health priority.

At the same time, digital technologies and social media platforms have become increasingly integrated into daily life and social communication. Social media may provide opportunities for older adults to maintain relationships with family members and friends, participate in online communities, access emotional support, and obtain health-related information. Digital engagement may be especially valuable for older adults experiencing geographic isolation, physical limitations, or reduced social participation. Prior studies suggest that online social engagement may improve perceived connectedness and reduce perceived social isolation for some older adults by facilitating communication and maintaining social ties (13–17). Prior research suggests that the relationship between social media use and perceived social isolation among older adults is mixed (18). Social media may reduce perceived social isolation by helping older adults maintain contact with family and friends, participate in online communities, access emotional support, and sustain social ties despite mobility limitations or geographic separation (15, 19–21). However, social media use may also be associated with higher perceived social isolation when engagement is primarily passive, when online interaction lacks reciprocity or emotional depth, or when users experience social comparison, misinformation exposure, negative content, or displacement of offline social contact (22, 23). The direction of this relationship may also be bidirectional: older adults with greater perceived social isolation may be more likely to use social media to seek connection, support, or distraction, while some forms of social media engagement may reinforce feelings of disconnection.

However, social media engagement may also produce unintended negative consequences for emotional well-being. Excessive social media exposure has been associated with social comparison, emotional exhaustion, misinformation exposure, fragmented communication, and reduced face-to-face social interaction (24–27). Passive forms of online engagement, such as prolonged browsing or video consumption without reciprocal interaction, may contribute to feelings of social disconnection despite increased digital activity. Older adults with limited digital literacy may face additional challenges navigating rapidly evolving online environments and interpreting online content (28, 29). Consequently, the relationship between social media engagement and perceived social isolation among older adults remains complex and potentially bidirectional.

These concerns may become increasingly important within emerging AI-mediated digital environments. Following the rapid expansion of generative artificial intelligence technologies after 2022, online communication and information ecosystems have become increasingly shaped by AI-enhanced recommendation systems, conversational agents, personalized content delivery algorithms, and automated digital interactions. AI-powered systems increasingly influence the type, frequency, and emotional tone of online content encountered by users, potentially affecting emotional well-being and perceptions of social connectedness. At the same time, disparities in digital access, technological literacy, and online navigation skills may further exacerbate existing social and health inequalities among aging populations. Despite growing attention to artificial intelligence and digital health, limited nationally representative research has examined how distinct social media engagement behaviors are associated with perceived social isolation among older adults. Several related digital concepts should be distinguished. Social media use refers to engagement with social networking platforms and includes behaviors such as visiting platforms, sharing content, interacting with others, and watching videos. General internet use is broader and includes online activities beyond social media, such as searching for information, using email, accessing patient portals, shopping, or using telehealth services. AI-enhanced digital environments refer to contemporary online settings in which platforms may incorporate generative AI tools, conversational agents, automated content systems, or AI-supported communication features. Algorithmic content exposure refers more specifically to the ranking, recommendation, filtering, or personalization of content by platform systems. These concepts overlap because social media platforms are internet-based environments that often rely on algorithmic systems and may increasingly incorporate AI-supported features. However, they are not equivalent. The present study directly measures social media engagement behaviors, not general internet use, AI use, or algorithmic exposure. Different forms of social media engagement may have distinct relationships with perceived social isolation. General platform visitation may reflect exposure to online social environments but does not necessarily involve meaningful interaction or social support. Sharing personal content may represent self-disclosure, identity expression, or attempts to maintain social ties, whereas sharing general content may reflect participation in broader information exchange without direct interpersonal connection. Interacting with others may be more reciprocal and socially oriented, but it may also occur because socially isolated individuals seek connection online. Watching videos may represent a more passive or consumption-oriented behavior that provides distraction or information but may offer limited interpersonal support. Therefore, examining these behaviors separately is important because active, passive, interpersonal, and content-consumption forms of engagement may differ in their associations with perceived social isolation among older adults.

Contemporary social media engagement increasingly occurs within algorithmically mediated digital environments. Long-standing platform features, including recommendation systems, personalized feeds, automated content ranking, and targeted content delivery, may shape what users see when they visit social media platforms, share personal or general content, interact with others, or watch videos. More recently, the public expansion of generative artificial intelligence has added new forms of conversational agents, AI-generated content, automated replies, and synthetic media to online communication environments. These developments may be particularly relevant for older adults, who may experience both benefits and challenges when navigating increasingly complex digital platforms. For example, algorithmic content delivery may help older adults encounter personally relevant information and social content, but it may also increase exposure to emotionally charged content, misinformation, social comparison, or passive browsing patterns. Similarly, AI-supported communication tools may lower barriers to online participation for some older adults, while also creating new challenges related to trust, digital literacy, and interpretation of online content. However, the HINTS 2022 and 2024 surveys do not directly measure AI use, AI-generated content exposure, or algorithmic recommendation exposure. Therefore, in this study, AI-enhanced digital environments are treated as contextual background, while the empirical analyses focus on five measured social media engagement behaviors: visiting social media, sharing personal content, sharing general content, interacting with others, and watching videos.

Therefore, this study used nationally representative HINTS 2022 and 2024 data to examine associations between multiple social media engagement behaviors and perceived social isolation among U.S. adults aged 65 years and older. Specifically, we examined five measured social media behaviors: visiting social media platforms, sharing personal content, sharing general content, interacting with others, and watching videos. Because HINTS do not directly measure AI use or algorithmic content exposure, references to AI-enhanced digital environments are used to contextualize the broader digital communication landscape rather than to indicate a directly measured exposure.

## 2 Materials and methods

### 2.1 Data source and study population

This study utilized pooled cross-sectional data from the Health Information National Trends Survey (HINTS) 7 cycles conducted in 2022 and 2024 by the National Cancer Institute. HINTS is a nationally representative survey designed to assess health communication behaviors, health information access, and technology use among adults in the United States. The analytic sample was restricted to adults aged 65 years and older. Participants with missing information on key study variables were excluded from the final analyses.

### 2.2 Outcome Variable

Perceived social isolation was measured using the HINTS-derived PROMIS Social Isolation Scale T-score (PROMIS_Isolation_t). This measure assesses subjective perceptions of social disconnection rather than objective social isolation or loneliness as a separate construct. The T-score was derived from four HINTS items: FeelLeftOut, FeelPeopleBarelyKnow, FeelIsolated, and FeelPeopleNotWithMe. These items assess whether respondents felt left out, felt that people barely knew them, felt isolated, and felt that people were not with them. Scores were converted to the standardized PROMIS T-score metric, with higher scores indicating greater perceived social isolation. Text-coded missing responses, including not ascertained, web partial, and blank responses, were treated as missing. The four source items demonstrated high internal consistency in the analytic sample (Cronbach's alpha = 0.887). The HINTS 2022 and 2024 surveys did not directly measure AI use, AI-generated content exposure, conversational AI use, or algorithmic recommendation exposure. Therefore, AI-related exposure was not operationalized as a study variable, and all references to AI-mediated or AI-enhanced digital environments are interpreted as contextual rather than directly measured. An overall social media engagement index was created from the five social media items: visiting social media platforms, sharing personal content, sharing general content, interacting with others, and watching videos. Each item was coded from 1 = Never to 5 = Almost every day, and the index was calculated as the mean of available nonmissing items. Higher scores indicate more frequent overall social media engagement. The five-item index demonstrated moderate internal consistency in the analytic sample (Cronbach's alpha = 0.698). Because the items capture related but distinct forms of engagement, the index was interpreted as a summary measure of overall social media engagement frequency rather than as a unidimensional psychosocial scale.

### 2.3 Social media engagement

Social media use was assessed using self-reported measures related to engagement with social networking platforms. Participants were categorized according to reported social media use frequency and engagement patterns based on available HINTS survey variables. Social media engagement was assessed using five self-reported HINTS items measuring how often respondents used social media in the following ways: visiting social media platforms, sharing personal content, sharing general content, interacting with others, and watching videos. Response categories were coded as 1 = Never, 2 = Less than once a month, 3 = A few times a month, 4 = At least once a week, and 5 = Almost every day. Text-coded missing responses, including not ascertained, web partial, and multiple responses selected in error, were treated as missing. Social media engagement was distinguished from general internet use, AI use, and algorithmic content exposure. The HINTS items analyzed in this study measured specific self-reported social media behaviors, including visiting social media platforms, sharing personal content, sharing general content, interacting with others, and watching videos. These variables did not measure general internet activity, direct use of AI tools, exposure to AI-generated content, or algorithmic recommendation exposure.

Social media behaviors were conceptually grouped into more passive/consumption-oriented and more active/participatory forms of engagement. Visiting social media platforms and watching videos were classified as more passive or consumption-oriented behaviors because they may involve exposure to online content without requiring direct reciprocal interaction. Sharing personal content, sharing general content, and interacting with others were classified as more active or participatory behaviors because they involve content production, self-disclosure, information sharing, or interpersonal exchange. However, HINTS does not directly measure passive browsing, such as scrolling through feeds or viewing others' posts without responding, nor does it assess reciprocity, emotional tone, platform type, or quality of online interaction. Therefore, this classification was used as a conceptual distinction based on available HINTS items rather than as a definitive taxonomy of social media behavior.

### 2.4 Covariates

Demographic and socioeconomic covariates included age, sex, race and ethnicity, educational attainment, and annual household income. Covariates were selected based on prior literature and conceptual relevance to both social media engagement and perceived social isolation among older adults. The main models adjusted for age, sex, race and ethnicity, educational attainment, household income, and survey cycle. Age was included because both technology use and perceived social isolation may vary across older age groups. Sex and race/ethnicity were included to account for demographic differences in digital engagement, social networks, and social resources. Educational attainment and household income were included as socioeconomic indicators that may shape digital access, digital literacy, online engagement, and opportunities for social participation. Survey cycle was included to account for differences between the 2022 and 2024 HINTS cycles.

### 2.5 Statistical analysis

All analyses incorporated HINTS survey weights to account for the complex sampling design and produce nationally representative estimates. Survey-weighted descriptive statistics were calculated to summarize participant characteristics. Multivariable regression analyses were conducted to examine the association between social media use and perceived social isolation after adjusting for demographic, socioeconomic, and digital access variables. Additional subgroup analyses were performed to explore potential differences across vulnerable populations, including older adults with lower educational attainment and limited digital literacy. Statistical significance was assessed using a two-sided significance level of *p* < 0.05. Data merging and cleaning were performed in R. All analyses were conducted in Stata 19.5.

The 2022 and 2024 HINTS cycles were pooled to increase the analytic sample size of adults aged 65 years and older and to improve precision for analyses of multiple social media engagement behaviors. Pooling was particularly important because several social media behavior variables had item-specific missingness and sparse responses in higher-frequency categories. The primary objective was to estimate overall associations between social media engagement and perceived social isolation among older adults across the recent HINTS 2022–2024 study period. To account for potential differences between survey years, survey cycle was included as an adjustment variable in multivariable models. In addition, we tested an interaction between survey cycle and overall social media engagement to assess whether the association differed between the 2022 and 2024 cycles. Because several high-frequency categories for active social media behaviors were sparse, sensitivity analyses were conducted using binary indicators for each social media behavior. For each item, respondents were categorized as reporting no engagement (“Never”) vs. any engagement (“Less than once a month” to “Almost every day”). These models assessed whether the direction and interpretation of the associations were robust to alternative coding of sparse ordinal exposure variables.

All analyses accounted for the complex HINTS sampling design using Stata survey procedures. The pooled final person-level weight was specified as the probability weight, and the HINTS design variables were used to account for stratification and clustering. Specifically, the survey design was declared in Stata using the svyset command with the pooled analytic weight, stratum variable, and cluster/primary sampling unit variable. Survey-weighted descriptive statistics and regression models were then estimated using the svy: prefix so that point estimates and standard errors reflected the HINTS sampling design. When pooling the 2022 and 2024 cycles, final person-level weights and replicate weights were divided by the number of pooled cycles, and a survey cycle indicator was included in adjusted models to account for differences between cycles.

Data from the 2022 and 2024 HINTS cycles were pooled to increase analytic sample size and improve precision. Because each HINTS cycle is independently weighted to represent the U.S. adult population for that survey year, we adjusted the survey weights by dividing them by the number of pooled cycles. Specifically, the pooled final person-level weight was calculated as person_finwt0/2, where 2 represents the number of HINTS cycles included. Replicate weights were adjusted using the same approach. This adjustment prevents the pooled dataset from representing twice the target population size while preserving the relative contribution of respondents within each cycle. All analyses accounted for the complex survey design using the pooled final weight, replicate weights, and survey design variables as appropriate. A survey cycle indicator was also included in adjusted models to account for differences between the 2022 and 2024 survey cycles.

Sociodemographic and digital access-related variables were included as covariates to adjust for differences in respondent characteristics. Interaction analyses were limited to age and survey cycle. Age was selected because the analytic sample was restricted to adults aged 65 years and older, and survey cycle was selected because the analysis pooled HINTS 2022 and 2024. These interaction analyses were exploratory and were used to assess whether the association between social media engagement and PROMIS social isolation T-scores varied across older age or between survey cycles. Adjusted models accounted for education and digital access-related characteristics. These factors were considered important covariates because lower educational attainment and limited digital literacy may shape both social media engagement and social isolation among older adults. However, we did not estimate formal subgroup-specific models by education or digital literacy; therefore, these findings should not be interpreted as subgroup effects.

### 2.6 Model assumptions and diagnostics

Model assumptions for linear regression were assessed using auxiliary diagnostic models. We examined residual-vs.-fitted plots to assess linearity, residual patterns, and potential influential observations. The residual plot did not suggest substantial nonlinearity or severe model misspecification. Multicollinearity was evaluated using variance inflation factors from unweighted auxiliary regression models because Stata does not support VIF estimation after svy: regression. No evidence of severe multicollinearity was observed; the mean VIF was 1.93, and all individual VIF values were below 5. These diagnostic models were used only to assess model assumptions. Final inference was based on survey-weighted linear regression models using Stata's svy: procedures to account for sampling weights, stratification, and clustering.

## 3 Results

### 3.1 Participant characteristics

The study sample consisted of older adults aged 65 years and older from the 2022 and 2024 cycles of the Health Information National Trends Survey (HINTS). The analytic sample was constructed through a stepwise selection process. The pooled HINTS 2022 and 2024 dataset included 13,531 respondents. The sample was first restricted to adults aged 65 years and older, resulting in 4,704 eligible respondents. Among these older adults, 157 were excluded because of missing PROMIS Social Isolation T-score data, leaving 4,547 respondents with nonmissing outcome data. An additional 1,236 respondents were excluded because of missing values for main covariates, including age, sex, race and ethnicity, education, household income, or survey cycle, resulting in 3,311 respondents with complete outcome and covariate data. Because each regression model examined a different social media engagement behavior, final analytic sample sizes varied across models due to item-specific missingness in social media variables. Final model-specific analytic samples ranged from 2,422 respondents for visiting social media to 3,248 respondents for interacting with others.

In Table 1, approximately half of the weighted sample was female (49.6%), and the majority identified as non-Hispanic White (74.0%), followed by Hispanic (10.1%) and non-Hispanic Black (9.5%) older adults. In terms of educational attainment, 41.7% reported some college education, while approximately 26.7% held at least a bachelor's degree. Household income levels varied across the sample, with 22.1% reporting annual incomes between $50,000 and $75,000 and 18.2% reporting incomes below $20,000. Participants from the 2024 survey cycle represented 55.8% of the analytic sample. The mean age of participants was 73.9 years (95% CI: 73.58–74.28). Perceived social isolation was measured using the PROMIS Social Isolation Scale T-score, with higher scores indicating greater perceived social isolation. The mean PROMIS Social Isolation T-score among older adults was 43.66 (95% CI: 43.27–44.06), suggesting moderate levels of perceived social isolation within the study population. Descriptive statistics for the analytic sample are presented in Table 1. Adjusted regression models included age, sex, race/ethnicity, education, household income, and survey cycle to account for demographic and socioeconomic differences. Because stratified descriptive analyses by demographic and socioeconomic groups were not presented, we do not interpret group differences in social media use descriptively in the Results section.

**Table 1:** Descriptive characteristics of U.S. Older Adults Included in the Study, HINTS 2022-2024.

Variable	n	Weighted % or Mean (95% CI)
Sex		
Male	2,386	50.4%
Female	2,346	49.6%
Race/Ethnicity		
Non-Hispanic White	3,243	74.0%
Non-Hispanic Black	415	9.5%
Hispanic	440	10.1%
Non-Hispanic Asian	121	2.8%
Non-Hispanic Other	162	3.7%
Educational Attainment		
Less than High School	400	8.4%
High School Graduate	1,103	23.2%
Some College	1,987	41.7%
Bachelor's Degree	632	13.3%
Post-Baccalaureate Degree	636	13.4%
Household Income		
<$20,000	675	18.2%
$20,000-<$35,000	689	18.6%
$35,000-<$50,000	718	19.4%
$50,000-<$75,000	821	22.1%
$75,000-<$100,000	260	7.0%
≥$100,000	549	14.8%
Survey Cycle		
2022 (2022 survey cycle)	2,395	44.2%
2024 (2024 survey cycle)	3,020	55.8%
Continuous Variables	n	Mean (95% CI)
Age	4,704	73.93 (73.58-74.28)
PROMIS Social Isolation T-score	4,872	43.66 (43.27–44.06)

Estimates were weighted using the HINTS complex survey design variables. Higher PROMIS Social Isolation Scale T-scores indicate greater perceived social isolation/social isolation.

### 3.2 Social media use among older adults

 Table 2 presents the distribution of social media engagement behaviors among older adults. Visiting social media platforms was relatively common, with 18.6% of older adults reporting social media use almost every day and 16.5% reporting use at least once a week, while 43.1% reported never visiting social media platforms. In contrast, active social media participation behaviors were substantially less common. Approximately 90.9% of older adults reported never sharing personal content on social media, and 79.6% reported never sharing general content. Similarly, 85.6% reported never interacting with others on social media platforms. Watching videos represented the most common passive social media behavior, with 57.3% reporting never watching videos, while approximately 42.7% reported some level of video engagement. Overall, the findings suggest that passive social media consumption behaviors were more prevalent than active content creation or interpersonal interaction among older adults during the study period.

**Table 2:** Frequency distribution of social Media engagement behaviors Among U.S. Older Adults, HINTS 2022–2024.

Frequency	Visited Social Media n (%)	Shared Personal Content n (%)	Shared General Content n (%)	Interacted With Others n (%)	Watched Videos n (%)
Never	1,752 (43.1%)	4,643 (90.9%)	4,093 (79.6%)	4,482 (85.6%)	2,881 (57.3%)
Less than once a month	420 (11.2%)	318 (5.9%)	761 (14.8%)	455 (9.7%)	1,343 (25.1%)
A few times a month	428 (10.5%)	122 (2.1%)	258 (3.9%)	178 (3.1%)	649 (12.1%)
At least once a week	652 (16.5%)	79 (1.0%)	105 (1.6%)	96 (1.5%)	284 (5.0%)
Almost every day	725 (18.6%)	7 (0.1%)	10 (0.1%)	11 (0.2%)	32 (0.5%)
Total	3,976 (100%)	5,168 (100%)	5,226 (100%)	5,221 (100%)	5,189 (100%)

Percentages represent survey-weighted proportions derived from the HINTS complex survey design. Missing responses, including not ascertained, web partial/question never seen, and multiple responses selected in error where applicable, were excluded from percentage calculations. Because each social media behavior item used its own valid-response denominator, columns should not be interpreted as conditional distributions among respondents who reported visiting social media. For sharing, interaction, and video-watching items, the “Never” category may include both respondents who do not visit social media and respondents who visit social media but do not engage in that specific behavior. Higher response categories indicate greater frequency of engagement in each social media behavior.

### 3.3 Association between social media use and perceived social isolation

As shown in Table 3, the pooled HINTS 2022 and 2024 dataset included 13,531 respondents, of whom 4,704 were aged 65 years and older. After excluding respondents with missing PROMIS Social Isolation T-score data and missing main covariates, 3,311 respondents had complete outcome and covariate data. Model-specific complete-case analytic samples ranged from 2,422 to 3,248 depending on the social media engagement variable examined. Survey-weighted multivariable linear regression analyses demonstrated consistent associations between multiple dimensions of social media engagement and higher perceived social isolation among older adults in the United States. Across all five models (Table 4), greater social media engagement was associated with significantly higher PROMIS Social Isolation Scale T-scores after adjusting for age, sex, race and ethnicity, educational attainment, and household income. More frequent general social media visitation was associated with higher perceived social isolation scores (*β* = 0.66, 95% CI: 0.37–0.95, *p* < 0.001). Similarly, sharing personal content on social media was positively associated with perceived social isolation (*β* = 1.15, 95% CI: 0.05–2.26, *p* = 0.040). Stronger associations were observed for sharing general content (*β* = 1.86, 95% CI: 0.81–2.91, *p* = 0.001) and interacting with others on social media platforms (*β* = 1.75, 95% CI: 0.79–2.71, *p* < 0.001). Watching videos on social media was also significantly associated with higher perceived social isolation scores (*β* = 1.13, 95% CI: 0.53–1.72, *p* < 0.001). Collectively, these findings suggest that the available HINTS indicators of social media engagement, including more participatory and more consumption-oriented behaviors, were associated with higher PROMIS social isolation T-scores among older adults.

**Table 3:** Sample selection process for the analytic sample, HINTS 2022–2024.

Selection step	n remaining	Additional n excluded at this step
Total respondents in pooled HINTS 2022 and 2024 sample	13,531	
Adults aged 65 years and older	4,704	8,827
Nonmissing PROMIS Social Isolation T-score	4,547	157
Nonmissing main covariates	3,311	1,236
Complete cases for Model 1: visiting social media	2,422	889
Complete cases for Model 2: sharing personal content	3,219	92
Complete cases for Model 3: sharing general content	3,237	74
Complete cases for Model 4: interacting with others	3,248	63
Complete cases for Model 5: watching videos	3,221	90

Missing responses were handled using model-specific complete-case analysis. Because each regression model examined a different social media engagement behavior, analytic sample sizes varied according to item-specific missingness in the exposure variable, outcome variable, and covariates. The “additional n excluded at this step” column refers to exclusions from the immediately preceding selection step.

**Table 4:** Survey-Weighted multivariable regression analyses examining associations between social Media engagement behaviors and PROMIS social isolation T-scores Among U.S. Older Adults, HINTS 2022–2024.

Variable	Model 1 *β* (95% CI)	Model 2 *β* (95% CI)	Model 3 *β* (95% CI)	Model 4 *β* (95% CI)	Model 5 *β* (95% CI)
Social media activity: Visiting social media	0.66*** (0.37, 0.95)				
Social media activity: Sharing Personal Content		1.15* (0.05, 2.26)			
Social media activity: Sharing General Content			1.86*** (0.81, 2.91)		
Social media activity: Interacting with Others				1.75*** (0.79, 2.71)	
Social media activity: Watching Videos					1.13*** (0.53, 1.72)
Age	0.04 (−0.04, 0.13)	−0.00 (−0.08, 0.07)	−0.01 (−0.08, 0.07)	−0.02 (−0.10, 0.06)	−0.00 (−0.08, 0.07)
Female	−0.94 (−2.05, 0.17)	−0.69 (−1.66, 0.29)	−0.66 (−1.62, 0.30)	−0.65 (−1.59, 0.30)	−0.50 (−1.45, 0.45)
NH Black	−0.66 (−2.12, 0.80)	−0.85 (−2.15, 0.45)	−1.03 (−2.35, 0.30)	−1.24 (−2.56, 0.08)	−1.59* (−2.92, −0.26)
Hispanic	−0.75 (−2.35, 0.86)	−1.13 (−2.64, 0.38)	−1.13 (−2.67, 0.41)	−1.15 (−2.68, 0.38)	−1.54 (−3.19, 0.11)
NH Asian	−0.36 (−5.09, 4.38)	0.54 (−3.55, 4.63)	0.51 (−3.66, 4.68)	0.43 (−3.78, 4.64)	0.00 (−4.30, 4.30)
NH Other	−1.35 (−4.50, 1.80)	−0.71 (−3.61, 2.19)	−0.75 (−3.66, 2.16)	−0.97 (−3.85, 1.90)	−1.02 (−4.04, 1.99)
HS Graduate	−2.99* (−5.51, −0.46)	−2.44 (−4.90, 0.02)	−2.64* (−5.07, −0.21)	−2.68* (−5.12, −0.24)	−2.71* (−5.21, −0.21)
Some College	−2.20 (−4.84, 0.44)	−1.46 (−4.00, 1.08)	−1.64 (−4.17, 0.90)	−1.63 (−4.16, 0.90)	−1.61 (−4.21, 1.00)
Bachelor's Degree	−1.39 (−4.00, 1.22)	−1.26 (−3.82, 1.30)	−1.56 (−4.13, 1.01)	−1.49 (−4.05, 1.07)	−1.71 (−4.34, 0.91)
Post-Baccalaureate Degree	−1.40 (−4.27, 1.48)	−1.11 (−3.79, 1.57)	−1.56 (−4.28, 1.16)	−1.49 (−4.22, 1.24)	−1.26 (−4.07, 1.55)
Income $20k-<$35k	−0.40 (−2.11, 1.32)	−0.13 (−1.78, 1.52)	−0.52 (−2.16, 1.11)	−0.25 (−1.88, 1.38)	−0.05 (−1.69, 1.60)
Income $35k-<$50k	−1.40 (−3.13, 0.33)	−0.98 (−2.60, 0.64)	−1.13 (−2.74, 0.48)	−1.06 (−2.65, 0.53)	−1.06 (−2.67, 0.54)
Income $50k-<$75k	−1.76* (−3.42, −0.10)	−1.52 (−3.12, 0.08)	−1.47 (−3.07, 0.14)	−1.34 (−2.93, 0.25)	−1.44 (−3.06, 0.19)
Income $75k-<$100k	−2.30* (−4.50, −0.10)	−1.75 (−3.93, 0.44)	−1.90 (−4.03, 0.24)	−1.79 (−3.98, 0.41)	−2.14* (−4.23, −0.04)
Income ≥$100k	−3.65** (−5.98, −1.33)	−3.33** (−5.35, −1.31)	−3.51** (−5.54, −1.48)	−3.44** (−5.40, −1.48)	−3.58*** (−5.52, −1.64)
Observations	2,395	3,188	3,207	3,218	3,191
R^2^	0.037	0.025	0.037	0.034	0.036

Survey-weighted linear regression models adjusted for age, sex, race/ethnicity, education, and household income. Outcome variable was the PROMIS Social Isolation Scale T-score, with higher scores indicating greater perceived social isolation/social isolation. Reference groups included male sex, non-Hispanic White race/ethnicity, less than high school education, and household income <$20,000. *p < 0.05, **p < 0.01, ***p < 0.001. The main social media predictor in each model was the ordinal frequency of the behavior named in the model heading, coded from 1 = Never to 5 = Almost every day. Higher values indicate more frequent engagement. Sample sizes vary across models because each model included a different social media engagement variable and was estimated using complete-case analysis for the outcome, exposure, and covariates. Differences in n reflect item-specific missingness and survey skip patterns in HINTS.

Sociodemographic differences were also observed across the models. Compared with non-Hispanic White older adults, non-Hispanic Black older adults generally reported lower perceived social isolation scores, with statistically significant differences observed in the model examining social media video consumption (*β* = −1.59, 95% CI: −2.92 to −0.26, *p* = 0.019). Hispanic older adults also tended to report lower levels of perceived social isolation across several models, although most associations did not reach statistical significance. Higher educational attainment was generally associated with lower perceived social isolation scores, particularly among older adults who completed high school, while higher household income demonstrated a clear inverse relationship with perceived social isolation. Compared with individuals reporting annual household incomes below $20,000, those with incomes of $100,000 or greater consistently exhibited significantly lower perceived social isolation scores across all models (all *p* < 0.01). Moderate reductions in perceived social isolation were also observed among some middle-income categories. Age and sex were not significantly associated with perceived social isolation across most models after adjustment for other covariates. Overall, the findings suggest that social media engagement among older adults may coexist with persistent or elevated feelings of perceived social isolation despite increased digital connectivity.

Sensitivity analyses using binary indicators of social media engagement are presented in (Table 5). Across all five behaviors, any social media engagement was significantly associated with higher PROMIS Social Isolation T-scores compared with no engagement. Any visiting of social media platforms was associated with a 1.85-point higher PROMIS Social Isolation T-score (95% CI: 0.89–2.81, *p* < 0.001). Any sharing of personal content was associated with a 1.63-point higher score (95% CI: 0.56–2.70, *p* = 0.003), while any sharing of general content was associated with a 2.08-point higher score (95% CI: 1.27–2.89, *p* < 0.001). Any interaction with others on social media was associated with a 2.73-point higher score (95% CI: 1.76–3.70, *p* < 0.001), and any video watching was associated with a 2.63-point higher score (95% CI: 1.86–3.40, *p* < 0.001). These findings were consistent in direction with the primary ordinal models, suggesting that the observed associations were robust to alternative coding of sparse social media frequency categories. The models had modest explanatory power, with R^2^ values indicating that only a limited proportion of variation in PROMIS social isolation T-scores was explained by social media engagement and covariates. These findings suggest that social media engagement is associated with social isolation, but that social isolation is likely shaped by additional psychosocial, health, household, and community-level factors not captured in the present models.

**Table 5:** Sensitivity analyses using binary social Media engagement indicators.

Social media engagement variable	n	*β*	95% CI	*p*-value
Any visiting social media vs. never	5,352	1.85	0.89–2.81	<0.001
Any sharing personal content vs. never	8,844	1.63	0.56–2.70	0.003
Any sharing general content vs. never	8,860	2.08	1.27–2.89	<0.001
Any interacting with others vs. never	8,875	2.73	1.76–3.70	<0.001
Any watching videos vs. never	8,722	2.63	1.86–3.40	<0.001

Each row represents a separate survey-weighted linear regression model. The outcome was the PROMIS Social Isolation T-score. Binary social media indicators compared any engagement (“Less than once a month” to “Almost every day”) with no engagement (“Never”). Models adjusted for age, sex, race and ethnicity, education, household income, and survey cycle.

### 3.4 Interaction effects

Survey-weighted multivariable regression analyses (Table 6) demonstrated a significant positive association between overall social media engagement and perceived social isolation among older adults in the United States. In the primary adjusted model, greater social media engagement was associated with higher PROMIS Social Isolation Scale T-scores after controlling for age, sex, race and ethnicity, educational attainment, household income, and survey cycle. Specifically, each one-unit increase in the overall social media engagement index was associated with an approximately 2.60-point increase in PROMIS social isolation scores (*β* = 2.60, 95% CI: 1.70–3.49, *p* < 0.001). The interaction between survey cycle and overall social media engagement was not statistically significant, suggesting that the association between social media engagement and perceived social isolation did not differ significantly between the 2022 and 2024 HINTS cycles. Similarly, the interaction model examining moderation by survey cycle indicated that the relationship between social media engagement and perceived social isolation did not significantly differ between 2022 and 2024 (interaction *β* = 0.93, 95% CI: −0.74 to 2.60, *p* = 0.273). The age interaction model also showed no statistically significant interaction between age and social media engagement (interaction *β* = −0.05, 95% CI: −0.16 to 0.05, *p* = 0.316), suggesting that the association between social media engagement and perceived social isolation remained relatively consistent across older age groups.

**Table 6:** Survey-Weighted multivariable and interaction regression models examining associations between overall social Media engagement and perceived social isolation Among U.S. Older Adults, HINTS 2022–2024.

Variable	Model 1 Main Effects *β* (95% CI)	Model 2 Survey Cycle Interaction *β* (95% CI)	Model 3 Age Interaction *β* (95% CI)
Overall social media engagement	2.60*** (1.70, 3.49)	2.12*** (1.18, 3.06)	6.61 (−1.59, 14.81)
2024 (Emerging AI Environment)	0.14 (−0.95, 1.22)	−1.27 (−3.88, 1.35)	0.16 (−0.93, 1.25)
Social media × 2024 interaction		0.93 (−0.74, 2.60)	
Age	0.01 (−0.07, 0.09)	0.01 (−0.07, 0.08)	0.09 (−0.08, 0.26)
Age × social media interaction			−0.05 (−0.16, 0.05)
Female	−0.77 (−1.71, 0.17)	−0.74 (−1.68, 0.20)	−0.76 (−1.70, 0.17)
NH Black	−1.46* (−2.77, −0.16)	−1.52* (−2.83, −0.20)	−1.48* (−2.78, −0.17)
Hispanic	−1.42 (−2.92, 0.08)	−1.50 (−3.01, 0.02)	−1.41 (−2.91, 0.09)
NH Asian	0.29 (−3.85, 4.43)	0.26 (−3.92, 4.45)	0.31 (−3.80, 4.43)
NH Other	−1.03 (−3.86, 1.80)	−1.13 (−3.96, 1.71)	−0.93 (−3.75, 1.89)
HS Graduate	−3.05* (−5.44, −0.65)	−3.13* (−5.52, −0.73)	−2.98* (−5.36, −0.59)
Some College	−2.05 (−4.55, 0.45)	−2.12 (−4.62, 0.39)	−1.97 (−4.48, 0.53)
Bachelor's Degree	−2.00 (−4.54, 0.54)	−2.06 (−4.62, 0.49)	−1.93 (−4.45, 0.60)
Post-Baccalaureate Degree	−1.92 (−4.61, 0.76)	−1.94 (−4.63, 0.76)	−1.88 (−4.55, 0.79)
Income $20k-<$35k	−0.20 (−1.80, 1.40)	−0.19 (−1.79, 1.42)	−0.23 (−1.83, 1.36)
Income $35k-<$50k	−1.25 (−2.80, 0.29)	−1.24 (−2.79, 0.31)	−1.27 (−2.82, 0.28)
Income $50k-<$75k	−1.47 (−3.02, 0.09)	−1.49 (−3.04, 0.07)	−1.50 (−3.05, 0.04)
Income $75k-<$100k	−1.85 (−3.95, 0.26)	−1.91 (−3.99, 0.18)	−1.87 (−3.97, 0.24)
Income ≥$100k	−3.41** (−5.41, −1.41)	−3.46** (−5.46, −1.47)	−3.42** (−5.43, −1.42)
Observations	3,252	3,252	3,252
R^2^	0.052	0.053	0.053

Survey-weighted linear regression models adjusted for age, sex, race/ethnicity, education, and household income. Outcome variable was the PROMIS Social Isolation Scale T-score, with higher scores indicating greater perceived social isolation/social isolation. Model 1 presents the primary association between overall social media engagement and perceived social isolation. Model 2 examines whether survey cycle moderated this association, while Model 3 examines whether age moderated the relationship between social media engagement and perceived social isolation. Reference groups included male sex, non-Hispanic White race/ethnicity, less than high school education, and household income <$20,000. *p < 0.05, **p < 0.01, ***p < 0.001.

Several sociodemographic factors were associated with perceived social isolation across the models. Compared with non-Hispanic White older adults, non-Hispanic Black older adults consistently reported significantly lower PROMIS social isolation scores across all three models (all *p* < 0.05). Hispanic older adults also tended to report lower perceived social isolation scores, although these associations were marginally significant in some models. Higher educational attainment was generally associated with lower levels of perceived social isolation, particularly among individuals who completed high school. In addition, household income demonstrated a consistent inverse relationship with perceived social isolation. Compared with older adults reporting annual household incomes below $20,000, individuals with household incomes of at least $100,000 exhibited significantly lower PROMIS social isolation scores across all models (all *p* = 0.001). Age and sex were not significantly associated with perceived social isolation after adjustment for other covariates. Overall, the findings suggest that greater social media engagement was associated with higher perceived social isolation among older adults regardless of survey cycle or age subgroup during the emerging AI-mediated digital environment period.

**Figure 1:**
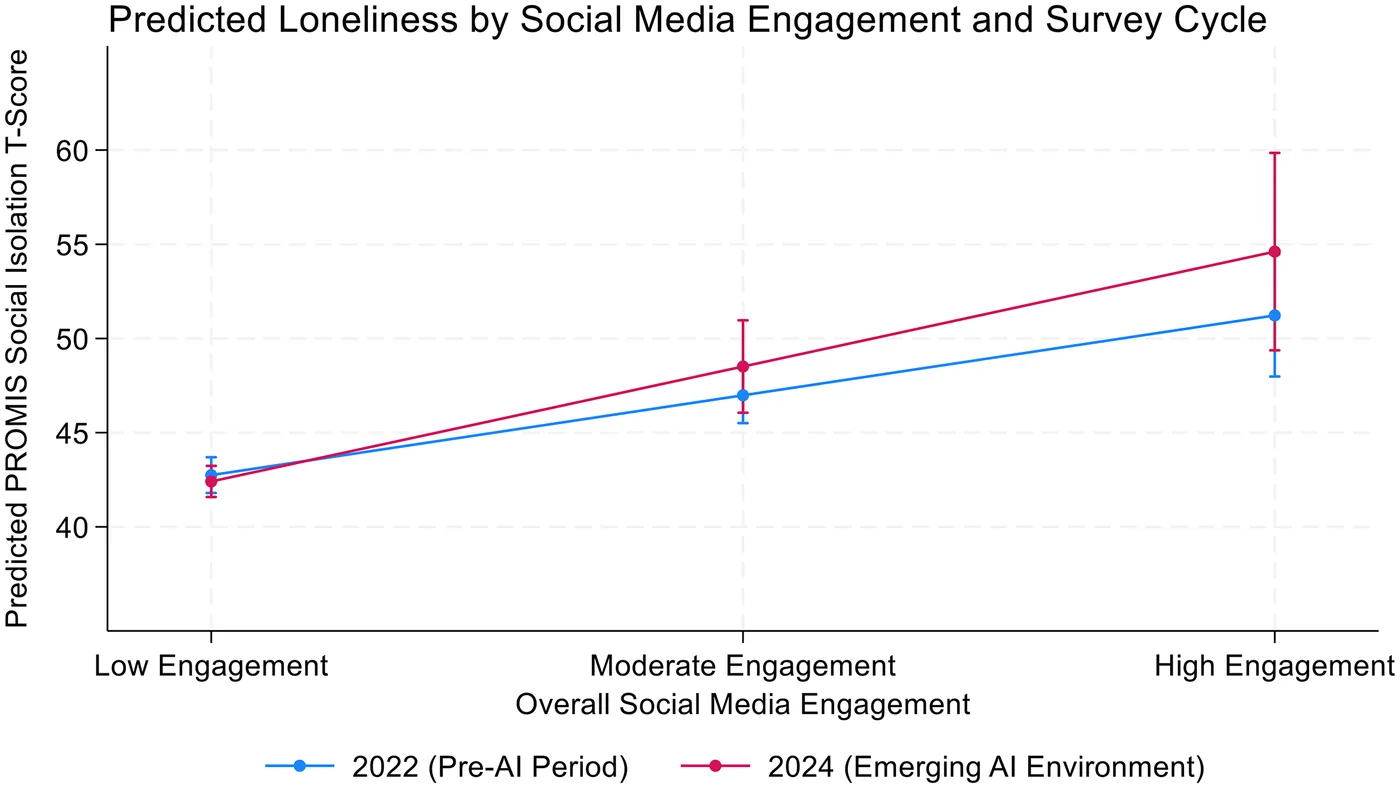
Predicted PROMIS social isolation T-scores by overall social Media engagement and survey cycle Among U.S. Older Adults, HINTS 2022-2024.

 Figure 1 illustrates adjusted predicted PROMIS Social Isolation T-scores across levels of overall social media engagement, stratified by HINTS survey cycle. In both 2022 and 2024, predicted perceived social isolation scores increased with greater social media engagement. Although predicted scores appeared slightly higher in 2024 at moderate and high engagement levels, the interaction between survey cycle and social media engagement was not statistically significant. Therefore, the figure suggests similar associations between social media engagement and perceived social isolation across the two survey cycles.

**Figure 2:**
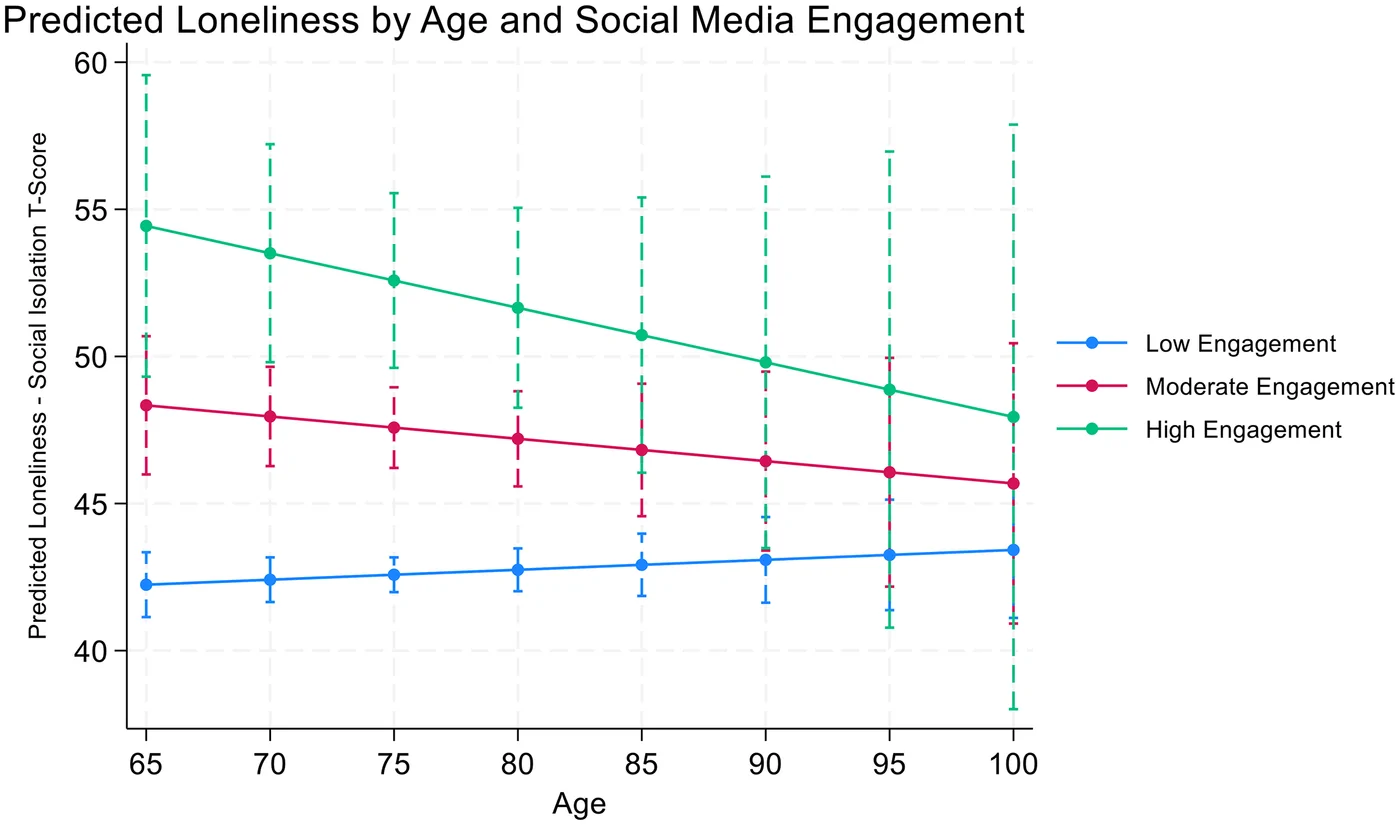
Predicted PROMIS social isolation T-scores by Age and overall social Media engagement Among U.S. Older Adults, HINTS 2022-2024.

 Figure 2 presents predicted perceived social isolation scores across older age groups according to levels of overall social media engagement. Older adults with high social media engagement consistently demonstrated the highest predicted PROMIS social isolation scores across nearly all age categories, whereas those with low social media engagement exhibited the lowest perceived social isolation scores. The figure also suggests modest age-related declines in predicted perceived social isolation among individuals with moderate and high social media engagement, while perceived social isolation levels among individuals with low engagement remained relatively stable across age groups. However, the interaction between age and social media engagement was not statistically significant in the adjusted regression model, indicating that the association between social media engagement and perceived social isolation did not substantially differ across older age groups.

## 4 Discussion

This study explored how different aspects of social media use relate to perceived social isolation among older adults in the U.S. during the early AI-influenced digital era, using data from the nationally representative HINTS 2022–2024 surveys. Key findings include: first, increased overall social media engagement was consistently associated with higher PROMIS Social Isolation T-scores among older adults, even after accounting for demographic and socioeconomic factors. Second, both active behaviors (like sharing content and engaging with others) and passive activities (such as browsing and watching videos) were positively associated with perceived social isolation. The distinction between passive and active social media engagement is important for interpreting these findings. Passive, consumption-oriented behaviors, such as visiting social media platforms or watching videos, may expose older adults to online content without necessarily providing reciprocal social support. Active behaviors, such as sharing content or interacting with others, may offer opportunities for connection and self-expression, but they may also reflect compensatory use among individuals who already experience perceived social isolation. Therefore, active engagement should not automatically be interpreted as protective, particularly in cross-sectional data. Third, this relationship remained stable across the 2022 and 2024 survey years, indicating that the link persisted through the initial growth phase of the evolving digital information environment. Lastly, higher education and household income were generally associated with less perceived social isolation, underscoring the ongoing influence of socioeconomic status on social well-being in older adults.

The finding that increased social media engagement correlates with higher perceived social isolation aligns with previous research indicating that digital connectivity doesn't always lead to meaningful social bonds among older adults (30). Although social media platforms are often promoted as tools for communication, social support, and connectedness, several studies have demonstrated that excessive or passive engagement may contribute to feelings of social comparison, emotional exhaustion, and perceived isolation (31, 32). Older adults who often use social media might see highly curated portrayals of others' lives, potentially heightening feelings of exclusion or social inadequacy. Additionally, passive behaviors like scrolling through content or watching videos without engaging in reciprocal interactions may offer few chances for genuine emotional support, despite more time spent online.

Interestingly, the study revealed that active behaviors on social media, such as sharing content and interacting with others, were linked to higher perceived social isolation levels. This may suggest that older adults who feel lonely are more likely to turn to digital platforms for social interaction or emotional support. In this light, social media use might serve as a way to cope rather than directly cause perceived social isolation. Since the study is cross-sectional, it cannot determine causality, and the relationship between social media and perceived social isolation is probably bidirectional. Older adults feeling socially isolated might rely more on social media for connection, while heavy use could also deepen feelings of disconnection from offline social networks.

The lack of a significant interaction between survey cycle and social media engagement suggests that the association between social media engagement and perceived social isolation did not differ significantly between the 2022 and 2024 HINTS cycles. This finding should not be interpreted as evidence that AI-related platform changes did or did not affect perceived social isolation, because AI use, AI-generated content exposure, and algorithmic recommendation exposure were not directly measured. Instead, the stable association across survey cycles may reflect broader and persistent patterns in how socially isolated older adults engage with social media. Long-standing recommendation systems and personalized feeds may shape online content exposure, while newer generative AI tools may introduce additional forms of automated communication and AI-generated content. Because HINTS did not measure either algorithmic exposure or AI exposure directly, future studies should examine these mechanisms using direct measures.

The study also identified several important sociodemographic patterns. Higher educational attainment and household income were generally associated with lower perceived social isolation scores, which aligns with prior research linking socioeconomic resources to improved social connectedness, communication access, and mental well-being (33–36). Individuals with greater educational attainment may possess stronger digital literacy skills and broader social support networks that facilitate healthier forms of online engagement. Similarly, higher-income older adults may have greater opportunities for social participation, healthcare access, transportation, and digital resources that reduce vulnerability to social isolation. The finding that non-Hispanic Black older adults reported lower perceived social isolation scores compared with non-Hispanic White older adults across several models may reflect differences in social support structures, family networks, or community engagement patterns, although these mechanisms could not be directly evaluated in the present study.

These findings have several public health implications. As healthcare systems and public health groups push for increased digital health communication and online activity among older adults, it's crucial to understand that greater digital engagement doesn't always boost psychosocial well-being. Interventions aiming to combat perceived social isolation should focus on facilitating meaningful and supportive social interactions rather than just increasing digital activity. Programs promoting community involvement, intergenerational dialogue, digital literacy, and healthy online habits can help older adults gain benefits from digital tools while reducing potential psychosocial risks. Additionally, the rise of AI-driven communication tools in social media and online health platforms necessitates careful observation of how algorithm-driven digital environments impact emotional health in aging populations.

This study has several limitations. First, its cross-sectional design limits the ability to draw causal conclusions about the relationship between social media engagement and perceived social isolation. Second, all data were self-reported, which may introduce recall bias or social desirability bias. Third, the HINTS dataset lacks detailed information on the quality, context, or specific platforms related to social media use. Different types of online engagement might have distinct psychosocial effects that the current analysis could not assess. The classification of social media behaviors as passive or active was limited by the available HINTS measures. Passive browsing, such as scrolling through feeds or viewing others' posts without responding, was not directly measured. Similarly, the survey did not capture the quality, emotional tone, reciprocity, or platform context of online interactions. As a result, the study may not fully capture important passive and active engagement patterns that could be associated with perceived social isolation. Fourth, although the models adjusted for key demographic and socioeconomic covariates, residual confounding may remain. Factors such as marital status, living arrangement, health status, disability, caregiving responsibilities, bereavement, offline social support, and quality of social relationships may influence both social media engagement and perceived social isolation but were not fully captured in the main models. Household income may also partly reflect household composition. For example, older adults with higher family income may be more likely to live with a spouse or partner, which could reduce perceived social isolation independently of digital engagement. Because marital status and living arrangement were not fully modeled, residual confounding by household structure remains possible. Finally, although the study is framed within the broader context of evolving AI-enhanced digital environments, HINTS did not directly measure AI use, AI-generated content exposure, conversational AI use, or algorithmic recommendation exposure. As a result, AI-related interpretations should be considered contextual and hypothesis-generating rather than direct empirical findings. Although the regression models identified statistically significant associations between social media engagement and PROMIS social isolation T-scores, the R^2^ values were modest, indicating that the models explained only a limited proportion of variation in social isolation. This is expected given that social isolation is a complex psychosocial outcome influenced by many factors not fully captured in HINTS, including living arrangement, caregiving status, social support, physical functioning, chronic disease burden, bereavement, neighborhood context, and offline social networks. Therefore, the findings should be interpreted as evidence of population-level associations rather than as a comprehensive predictive model of social isolation. Future studies incorporating richer measures of social networks, household structure, health status, and longitudinal changes in technology use may improve explanatory power.

Despite these limitations, the research offers important nationally representative insights into social media use and perceived social isolation among older adults amidst rapid digital transformation. The results indicate that higher social media engagement is consistently linked with increased perceived social isolation across various aspects of digital behavior. As AI-mediated communication continues to develop, further longitudinal and mixed-method studies are necessary to deepen understanding of how digital interaction patterns impact emotional well-being, social connectedness, and healthy aging among older populations.

## 5 Conclusion

Using nationally representative HINTS 2022–2024 data, this study found that greater social media engagement was consistently associated with higher levels of perceived social isolation among U.S. older adults. Both active and passive forms of social media engagement, including content sharing, interpersonal interaction, social media visitation, and video consumption, demonstrated positive associations with PROMIS Social Isolation T-scores. The findings further suggest that these relationships remained relatively stable during the emerging AI-mediated digital environment period. Although digital technologies may provide opportunities for communication and information access, increased online engagement alone may not adequately address social isolation among aging populations. Public health interventions and digital health strategies should therefore prioritize meaningful social connection, supportive communication environments, and digital literacy among older adults. As AI-driven digital ecosystems continue to expand, future research should examine how algorithmically mediated online experiences influence emotional well-being, social connectedness, and healthy aging trajectories over time.

## Data Availability

Publicly available datasets were analyzed in this study. This data can be found here: https://hints.cancer.gov/.
